# Establishment and Validation of Extra-transitional Zone Prostate Specific Antigen Density (ETzD), a Novel Structure-based Parameter for Quantifying the Oncological Hazard of Prostates with Enlarged Stroma

**DOI:** 10.1038/s41598-018-36602-x

**Published:** 2019-01-25

**Authors:** Jung Jun Kim, Yoon Seok Suh, Tae Heon Kim, Seong Soo Jeon, Hyun Moo Lee, Han Yong Choi, Seonwoo Kim, Kyu-Sung Lee

**Affiliations:** 1 Department of Urology, Seoul National University Bundang Hospital, Seongnam, Republic of Korea; 2 Department of Urology, Samsung Medical Center, Sungkyunkwan University School of Medicine, Seoul, Korea; 3 Statistics and Data Center, Samsung Medical Center, Seoul, Korea; 4 Department of Medical Device Management and Research, SAIHST, Sungkyunkwan University, Seoul, Korea

## Abstract

Extra-transitional zone density (ETzD), a novel parameter is proposed to stratify the deviation of prostate specific antigen (PSA) due to structural change according to stromal hyperplasia of prostate. ETzD was conducted on a concept to estimate the PSA density (PSAD) after hypothetical enucleation of the transitional zone of an enlarged prostate by a non-linear regression prediction model with intrinsic linearity, from the retrospective analysis of PSA change observed actual enucleation by laser. The performance to predict the presence and severity of malignancy was validated by two cohorts of 3,440 prostate biopsies and 2,783 radical prostatectomy specimens. The performance of ETzD was compared with conventional parameters. The receiver operative curve of area under curve (AUC) of ETzD to predict the presence of malignacy was 0.862 (95% CI; 0.843~0.881), better than PSA, PSAD or transitional zone PSAD (TzPSAD). The AUC of ETzD to predict an unfavorable cancer among prostate cancer patients was 0.736 (95% CI; 0.705~0.768), which performs better than PSA and comparable to PSAD or TzPSAD. In summary, the performance of ETzD as a universal parameter to quantify the oncological hazard of a prostate was validated and the superiority to conventional parameters was verified.

## Introduction

Serum Prostate Specific Antigen (PSA), considered as an important marker predicting prostate malignancy, has been clinically applied for prostate cancer screening and risk evaluation as well as follow-up marker after definite therapy for prostate cancer. PSA displays excellent performance as follow-up marker after treatment, but screening and risk evaluation for prostate cancer using PSA has been relatively less satisfactory. Elevation of PSA due to non-malignant conditions such as benign prostatic hypertrophy and prostatitis has emerged as an important limitation in oncologic hazard assessment^1^.

The contribution of enlarged prostate to serum PSA level in each patient serves as a barrier to precise interpretation of the oncological hazard of an enlarged prostate. Our study was initiated with the idea that estimating the impact of benign prostate enlargement on PSA was enabled by measuring post-operative changes of serum PSA levels after surgical removal of enlarged prostate adenoma, which represents transitional zone. Theoretically, transitional zone of prostate can be completely enucleated after Holmium Laser Enucleation of the Prostate (HoLEP). Based on an analysis of post-enucleation PSA changes in a HoLEP cohort, we developed a statistical model stratifying the impact of adenoma enlargement on PSA. We propose a stratified novel clinical parameter, Extra-Transitional zone PSA Density (ETzD), that reflects the oncological hazard of prostate by hypothetical nucleation of enlarged adenoma, which implies exclusion of the contribution of adenoma enlargement. The core element of this non-linear regression modeling is its intrinsic linearity between PSA and prostate size parameters measured by trans-rectal ultrasonography, whereas benign hyperplasia increases PSA level. As this novel parameter was designed for a substitute to conventional parameters representing prostate oncological hazards, our performance analysis included two functions of conventional clinical PSA measurement: detection of the presence of prostate cancer in screening and definition of the oncological status of prostate cancer patients before definitive treatment. First performance analysis consisted of evaluating the sensitivity and specificity of ETzD for detection of prostatic malignancy compared with TRUS-guided biopsy for prostate cancer detection among prostates with stromal hyperplasia. Second performance analysis consisted of evaluating the ability of ETzD to predict the severity of malignancy, essential information when identifying candidates for active surveillance. Performance of ETzD was compared with that of PSA, PSA density (PSAD) and transitional zone PSAD (TzPSAD).

## Materials and Methods

### Cohorts

Three different clinical cohorts were analyzed, one group for ETzD development and the other two groups for performance analysis. A HoLEP cohort was used to analyze the attribution of structural changes due to transitional zone enlargement to serum PSA level, leading to the development of ETzD. The ETzD concept was subsequently applied to a TRUS-guided biopsy cohort for performance analysis in predicting the presence of prostatic malignancy among prostates with stromal enlargement and moderately elevated PSA. The last cohort consisted of patients who underwent radical prostatectomy, and was used for a second performance analysis of ETzD to predict the oncological status of the prostate before definitive surgical treatment after a diagnosis of prostate cancer. This study including the retrospective review of three clinical cohorts was approved by the Institutional Review Board of the Samsung Medical Center and carried out in accordance with approved guidelines.

### ETzD development

#### HoLEP Cohort

ETzD was developed and validated based on multivariate non-linear regression analysis of clinical cohort of 383 pateints who underwent HoLEP surgery by single surgeon to treat benign prostate hyperplasia diagnosed by trans-rectal ultrasonography, as previously described^2^. We retrospectively reviewed patient records using prospectively maintained database. We created a novel formula estimating PSAD after hypothetical enucleation of enlarged adenoma, which could be considered transitional zone in TRUS. An assumption of equivalence between the transitional zone volume of preoperative TRUS and the actual enucleated prostate volume measured by surgery was validated. Cases exhibiting large variance of more than two standard deviations between the preoperative transitional zone size and enucleated weight were excluded.

Before development of final statistical model, we began with a preliminary linear regression model to predict the degree of PSA change by enucleation as intrinsic linearity inside final non-linear model. This preliminary linear model was derived from the pathophysiologic assumption that post-enucleation PSA change would exhibit constant linearity with preoperative PSA level and prostate size parameters, including total prostate size (PS), transitional zone size (TzS), and transitional zone index (TZI, the ratio of TzS and PS). To validate this assumption of linearity between enlarged prostate size and elevated PSA production, after preliminary confirmation based on univariate regression, multivariate linear regression model was used to predict the amount of PSA change by transitional zone enucleation of training cohort. Reproducibility of this multivariate linear regression model was evaluated for the validation cohort. By means of transformation of this intrinsic linear model to non-linear model using inverse relationship between size parameter and PSA, the following formula was created for estimating PSA per volume after hypothetical enucleation of transitional zone. This novel formula is intended to represent a stratified oncological hazard in excluding the impact of BPH on PSA: (Preoperative PSA – Estimated amount of PSA change by enucleation)/(PS – TzS).

#### Exclusion criteria

To analyze the pure influence of benign adenoma enlargement on PSA levels, data was collected from uninfected and nonmalignant prostate cases. All patients in cohort were evaluated for history, physical examination with digital rectal examination, urinalysis, and prostate-specific antigen (PSA) level at baseline to rule out infection or malignancy before proceeding to surgery. Cases of pathologically diagnosed prostate cancer in surgical specimens, persistent PSA elevation (≥4.0 ng/ml) 6 months after surgery or the discordance of predicted and actual resection volume (>2 standard deviation) were also excluded from our analysis.

### First ETzD performance analysis

#### Prostatic malignancy detection

Between May 2008 and October 2011, a total of 3,440 patients underwent a primary TRUS-guided prostatic biopsy at a single institution because of PSA elevation. Sensitivity, specificity and area under curve (AUC) of the ETzD ROC for the prediction of prostatic malignancy for enlarged prostate (≥30 cc) with moderately elevated PSA (2.5 to 20.0 ng/ml) before primary biopsy was evaluated and used to compare the performance of ETzD with that of PSA, PSAD and transitional zone PSAD (TzPSAD). This performance analysis served to validate the clinical efficacy of the new formula aimed at compensating for the contribution of benign transitional zone hypertrophy to PSA levels. ETzD performance was also compared to a linear regression prediction model based on data from the TRUS cohort only under the assumption of linearity between each parameter and prostate malignancy, including PSA, PS and TzS.

### Second ETzD performance analysis

#### Unfavorable prostate cancer prediction

Total of 3,237 patients underwent radical prostatectomy for prostate cancer between May 1994 and December 2011 at single institution. Among these patients, records were available for preoperative trans-rectal ultrasonography or prostate magnetic resonance imaging. These provided size information for the total prostate and transitional zone before radical prostatectomy, and were analyzed. ETzD performance was analyzed, including sensitivity, specificity, and AUC of the receiver operating characteristic (ROC) to predict unfavorable prostate cancer defined as high T stage (≥T3), high Gleason score sum (≥7), or positive lymph node (≥N1). In addition to evaluating the ETzD’s unfavorable cancer prediction performance, we also compared it to the PSA and PSAD. The performance to predict more than two factors constituting unfavorable cancer was also evaluated and compared.

### Statistical analyses

Descriptive statistics are presented as the mean ± standard deviation or median (interquartile range). Comparisons for continuous variables between any two groups were assessed with an independent t-test for normally distributed variables and Mann–Whitney’s U test for non-normally distributed variables. The normal distribution of the data was checked with Shapiro-Wilks test. Categorical variables were compared using the chi-square test. Pearson’s r was used to evaluate correlations between variables. Predictive performances were evaluated by receiver operating curve (ROC) analysis and AUCs were compared by DeLong method. The software used for statistical analysis was SPSS 20.0 (SPSS Inc, Chicago, IL, USA) with the R statistical package. All P-values < 0.05 were considered significant.

## Results

### ETzD development

**Figure 1:**
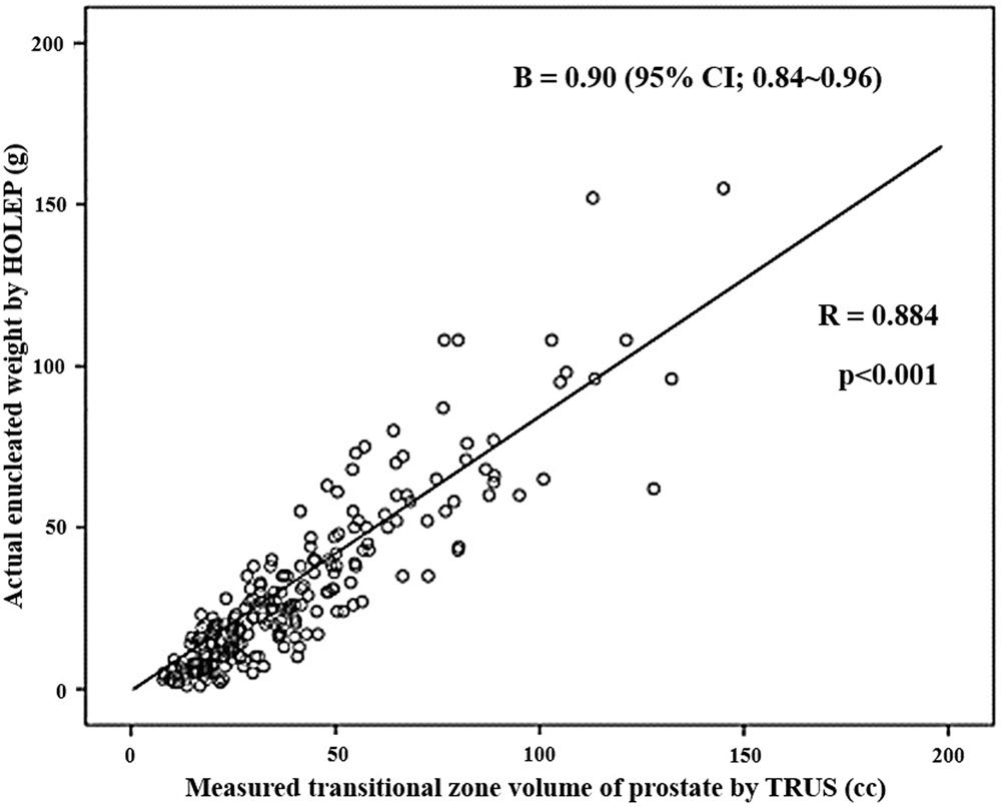
Scatterplot demonstrating the linear correlation between actual enucleated prostate weight by HoLEP and measured transitional zone volume by TRUS. Dots indicate frequency distribution of development cohort before exclusion (n = 254). HoLEP; Holmium Laser Enucleation of the prostate, TRUS; trans-rectal ultrasonography.

**Figure 2:**
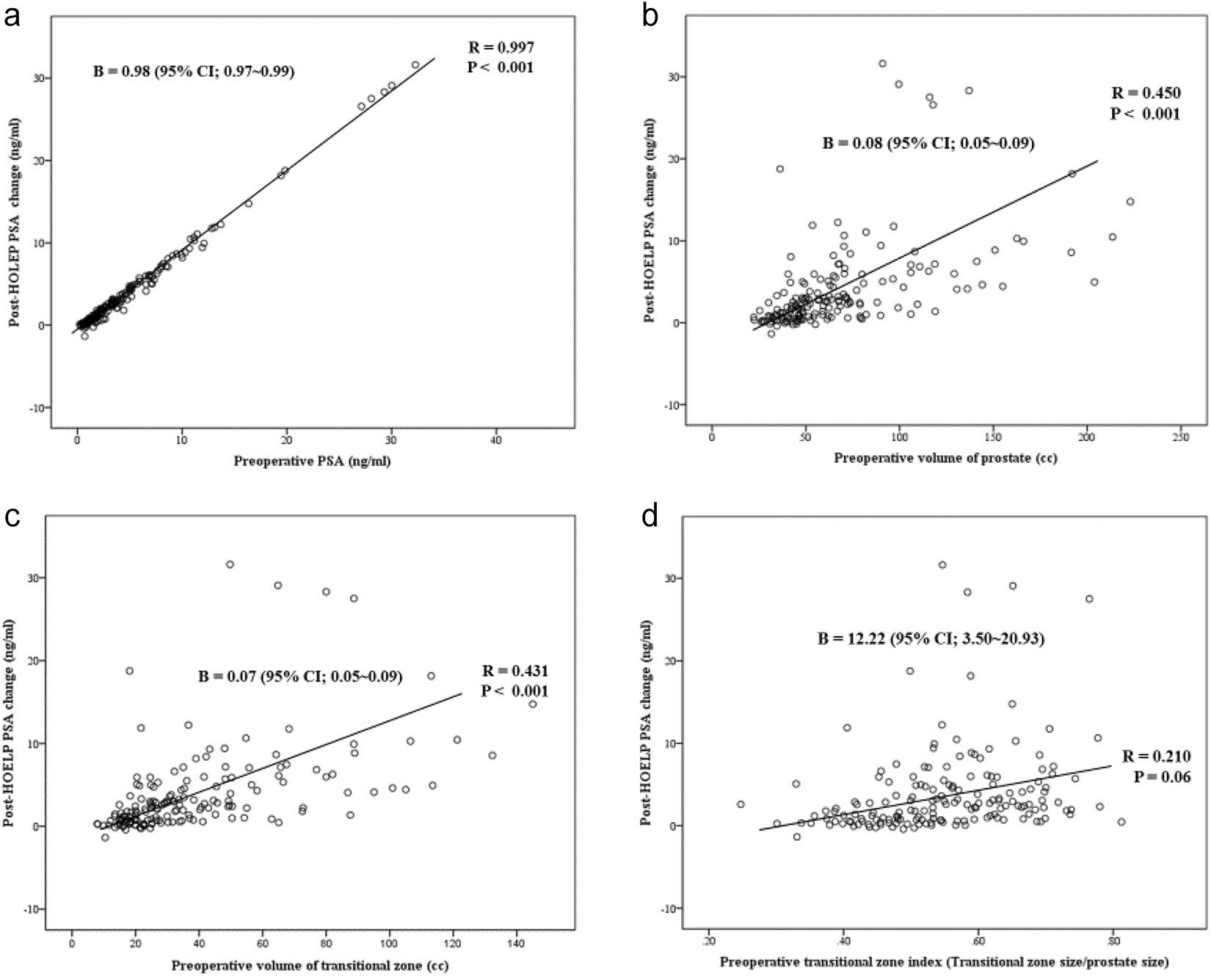
Scatterplot demonstrating the linear correlation between the degree of PSA change after enucleation of the transitional zone and (**a**) preoperative PSA, size parameters including (**b**) preoperative total volume of prostate measured by TRUS, (**c**) preoperative volume of transitional zone and (**d**) preoperative transitional zone index (TZI). Dots indicate frequency distribution of development cohort (n = 185). PSA; prostate specific antigen, TRUS; trans-rectal ultrasonography.

**Figure 3:**
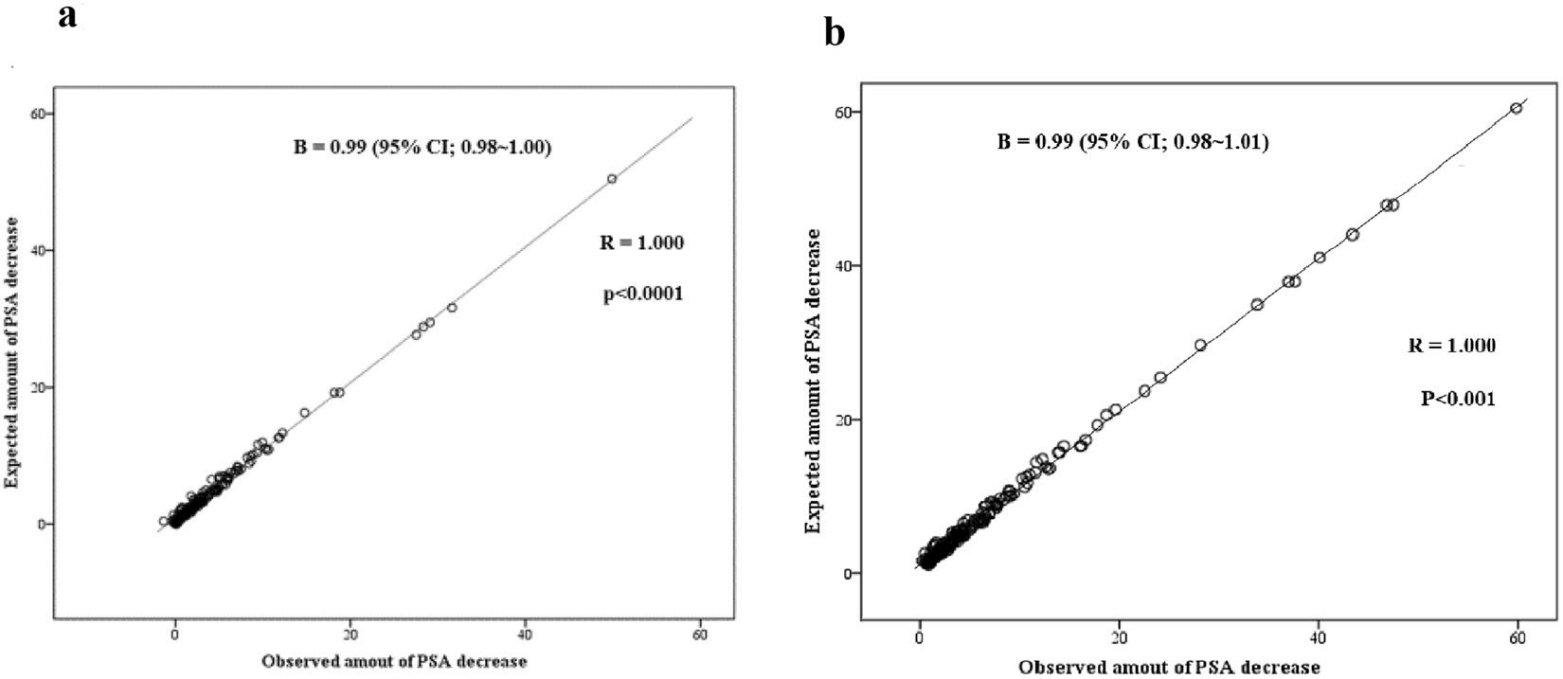
Scatterplot showing the observed linearity plot for predicted PSA decrease after enucleation by a multivariate linear regression model predicting actual data from post-HoLEP PSA among (**a**) training cohort (n = 185) and (**b**) validation cohort (n = 109). Dashed line indicates ideal predictions. Dots indicate frequency distribution of validation cohort. HoLEP; Holmium Laser Enucleation of the prostate, PSA; prostate specific antigen.

From cohort of 383 consecutive HoLEP cases performed between 2008 and 2015 (Table 1), initial 254 cases were classified as development cohort. The actual resected weight and preoperative transitional zone size by TRUS were correlated and comparable, given the vaporization nature of the Holmium laser (Fig. 1). Among development cohort, we excluded 69 patients: 54 due to follow-up loss, 12 due to prostate cancer diagnosis, 10 due to discordance between the predicted and actual resection volume, 4 due to persistent PSA elevation. PSA size parameters, including preoperative PSA, total PS, TzS and TzI, were correlated with the amount of PSA change after enucleation of the transitional zone. Both unidimensional linear and exponential correlations, as shown in Fig. 2 (n = 185), were consistent with the hypothesis that prostates with an enlarged adenoma produce more PSA. The multivariate linear regression model based on demonstrated a strong correlation with actual PSA change not only with development set (Fig. 3a) but also training set consists of another 129 HoLEP cases (Fig. 3b, n = 105, after exclusion of 19 follow-up loss, 4 discordances of predicted and actual resection volume and 2 prostate cancer). Based on this multivariate linear model predicting the amount of PSA from enlarged adenoma, a non-linear model of internal linearity was calculated to estimate the PSA contribution of prostatic areas outside the transitional zone per volume after the application of the concept of density. The PSA density outside the transitional zone, ETzD, was calculated as (1.068 + 0.016*PSA + 0.004*PS −1.02*TZI)/(PS − TzS).

**Table 1:** Patient characteristics for the Holium laser enucleation of prostate (HoLEP) cohort for

Characteristic	Total (n = 383) Mean ± SD
Patient age (year)	68.9 ± 7.5
BMI (kg/m^2^)	24.1 ± 3.0
**Size parameter (preoperative, by TRUS)**	
Total prostate volume (cc)	67.9 ± 36.5
Transitional zone volume (cc)	38.8 ± 25.4
**Weight of resected tissue (g)**	
Actual	29.1 ± 25.6
Estimated (considering 20% vaporization)	34.9 ± 30.7
**PSA (ng/mL)**	
Preoperative	5.5 ± 8.1
Postoperative 6 month	0.8 ± 0.5
**PSAD (ng/mL/cc)**	
Preoperative	0.081 ± 0.103
Postoperative 6 month	0.021 ± 0.023
Predicted post-enucleation PSA density (ETzD, ng/ml/cc)	0.021 ± 0.019

Development of Extra-Transitional zone Density (ETzD).

BMI; Body mass index, TRUS; Trans-rectal ultrasonography, PSA; Prostate specific antigen, PSAD; PSA Density.

### First ETzD performance analysis

**Figure 4:**
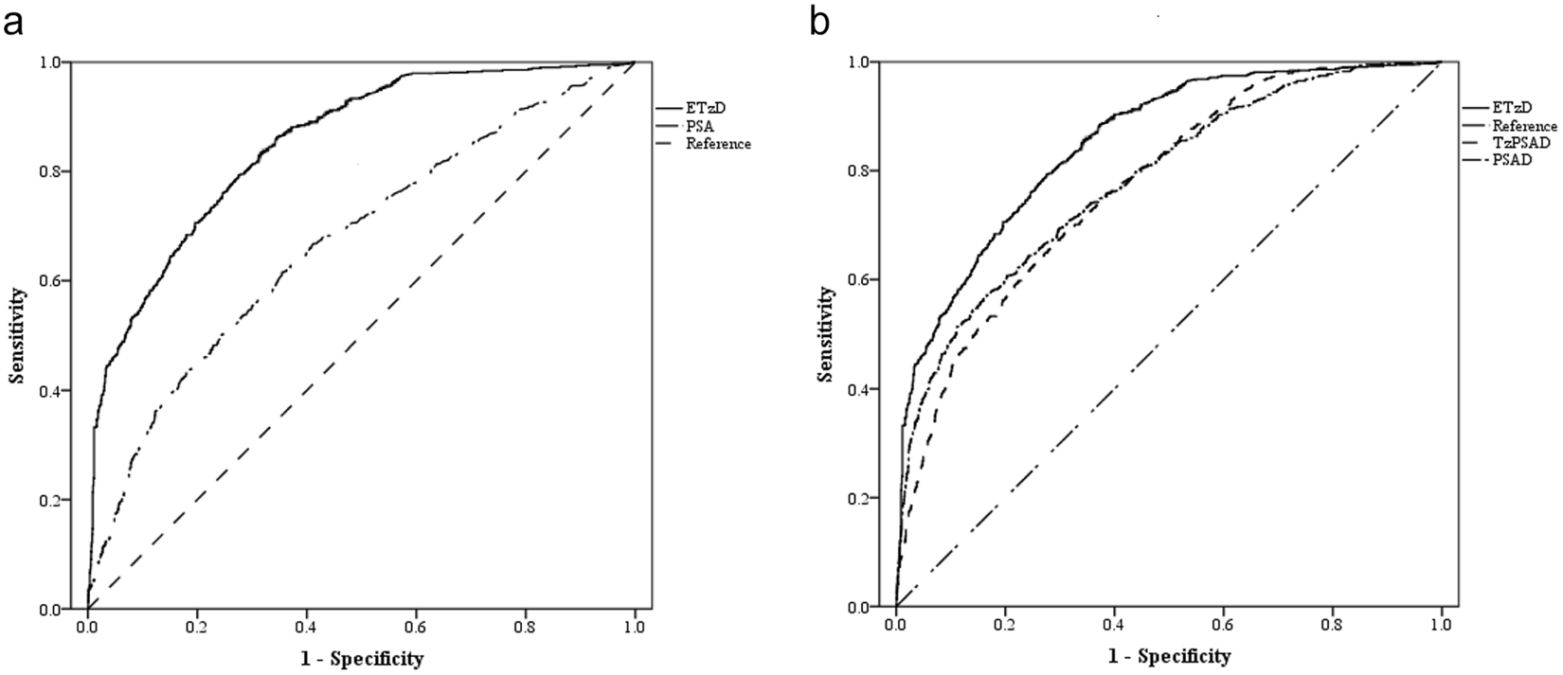
ROC curves for (**a**) ETzD with PSA and (**b**) ETzD with PSAD and TzPSAD for prostate cancer detection of TRUS-guided 12-core prostate biopsies. PSA; prostate specific antigen, PSAD; PSA Density, TzPSAD; transitional zone PSAD, ETzD; Extra-Transitional zone Density, TRUS; trans-rectal ultrasonography.

Demographic data and pathologic results obtained from our analysis of the primary 12-core TRUS biopsy database are listed in Table 2. Among 3,440 patients, 1,885 prostate biopsies for stromal enlargement with moderate PSA elevation were deemed appropriate for analysis. The AUC for ETzD predicting the presence of malignancy was 0.862 (95% CI; 0.843~0.881), which was higher than the AUC for PSA (0.672, 95% CI; 0.648~0.697, P = 0.001, Fig. 4a), PSAD (0.746, 95% CI; 0.724~768, P = 0.001, Fig. 4b) or TzPSAD (0.774, 95% CI; 0.754~795, Fig. 4b). The AUC of the ROC predicted by a conventional linear regression prediction model based on data obtained solely from the TRUS cohort was 0.766 (95% CI; 0.747~0.827), which was lower than that obtained with the ETzD (P = 0.001). A head–to-head comparison between ETzD and PSAD for malignancy detection predictive performance was conducted to identify a cut-off value for ETzD. The predictive performance of ETzD ≥ 0.1 exhibited higher specificity, positive predictive value, positive likelihood ratio and accuracy with a similar negative predictive value and sensitivity compare with both PSAD ≥ 0.15 and TzPSAD ≥ 0.35, as shown in Table 3.

**Table 2:** Characteristics for the 12-core TRUS-guided prostate biopsy cohort for predictive performance analysis of ETzD.

Parameter	Total Group	Negative Malignancy*	Positive Malignancy*	*P*-value (Negative vs Positive)
No. of patients	1885	1622	263	
Age (yr)	64.9 ± 8.5	64.5 ± 8.4	67.3 ± 8.0	<0.001
PSA (ng/mL)	5.85 ± 3.29	5.61 ± 3.00	7.35 ± 4.43	<0.001
**Prostate volume (cc)**				
Total	48.67 ± 19.88	50.22 ± 20.65	39.11 ± 9.91	<0.001
Transitional zone	23.44 ± 13.81	24.62 ± 14.19	16.17 ± 8.00	<0.001
Extratransitional zone	25.01 ± 7.81	25.35 ± 8.12	22.94 ± 5.20	<0.001
PSAD (mg/ml/cc)	0.130 ± 0.080	0.119 ± 0.063	0.195 ± 0.127	<0.001
TzPSAD (mg/ml/cc)	0.313 ± 0.258	0.279 ± 0.188	0.526 ± 0.454	<0.001
ETzD (mg/ml/cc)	0.056 ± 0.095	0.042 ± 0.008	0.142 ± 0.061	<0.001

PSA; prostate specific antigen, PSAD; PSA Density, TzPSAD; transitional zone PSAD, ETzD; Extra-Transitional zone Density. Data are presented as the mean ± SD.

*Definition of malignancy; the presence of a pathologically malignant adenocarcinoma from the specimen of 12-core TRUS-guided prostate biopsy.

**Table 3:** Head-to-head comparison of malignancy detection predictive performance between Extra-Transitional zone Density (ETzD), PSA density (PSAD) and transional zone PSA density (TzPSAD). The predictive performance of PSAD ≥ 0.15 was compared with ETzD ≥ 0.1 and TzPSAD ≥ 0.35, because each cut-off value demonstrated a similar negative predictive value.

Statistic	ETzD ≥ 0.1 ng/ml/cc		PSAD ≥ 0.15 ng/ml/cc		TzPSAD ≥ 0.35 ng/ml/cc		ETzD vs PSAD	ETzD vs TzPSAD
	Value	95% CI	Value	95% CI	Value	95% CI	p	p
Sensitivity	58.94%	52.73% to 64.94%	52.09%	45.87% to 58.27%	57.41%	51.19% to 63.47%	0.125	0.341
Specificity	84.22%	82.35% to 85.96%	77.56%	75.45% to 79.57%	76.63%	74.50% to 78.67%	<0.01	<0.01
Positive Likelihood Ratio	3.73	3.21 to 4.34	2.32	2.00 to 2.69	2.46	2.14 to 2.82	<0.01	<0.01
Negative Likelihood Ratio	0.49	0.42 to 0.56	0.62	0.54 to 0.70	0.56	0.48 to 0.64	0.08	0.251
Positive Predictive Value	37.71%	34.24% to 41.32%	27.35%	24.52% to 30.36%	28.49%	25.80% to 31.35%	<0.01	<0.01
Negative Predictive Value	92.67%	91.62% to 93.61%	90.90%	89.77% to 91.91%	91.73%	90.58% to 92.76%	0.11	0.412
Accuracy	80.69%	78.83% to 82.45%	74.01%	71.96% to 75.97%	73.95%	71.91% to 75.92%	<0.01	<0.01

### Second ETzD performance analysis

**Figure 5:**
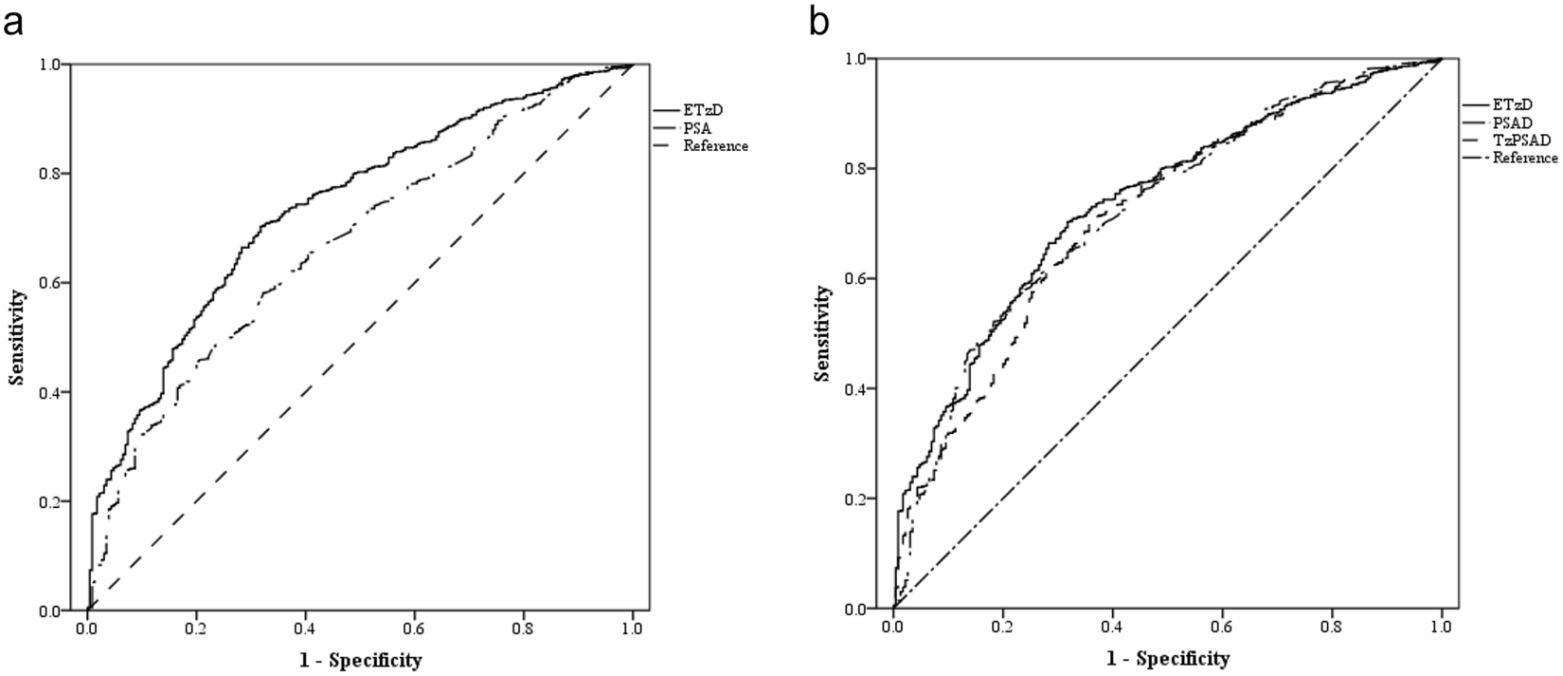
ROC curves of (**a**) ETzD with PSA and (**b**) ETzD with PSAD and TzPSAD for predicting unfavorable prostate cancer of surgical pathology with the definition of high pathological Gleason sum (≥7), extraprostatic extension (≥pT3), or nodal involvement. ETzD; Extra-Transitional zone Density, PSA; prostate specific antigen, PSAD; PSA Density, TzPSAD; transitional zone PSAD.

Among 3,237 patients who underwent radical prostatectomy, 2,783 records with preoperative imaging were available. The clinical characteristics of the patient group with preoperative imaging were not different from those of the total patient population (Table 4). The performance of ETzD to predict unfavorable prostate cancer of radical prostatectomy specimen among biopsy-proven prostate cancer patient is shown in Fig. 4. Among 2,783 patients, 2,548 (91.6%) were compatible with the definition of unfavorable prostate cancer. The clinical characteristics for patients with unfavorable and favorable prostate cancer are shown in Table 4. The AUC for ETzD was 0.736 (95% CI; 0.705~0.768), which was higher than that for PSA (0.692, 95% CI; 0.660~0.724, P = 0.04, Fig. 5a) and similar to that for PSAD (0.723, 95% CI; 0.690~0.757) or TzPSAD (0.714, 95% CI; 0.679~0.748, Fig. 5b).

**Table 4:** Baseline characteristics of a radical prostatectomy database for performance analysis to predict unfavorable prostate cancer.

Variable	Unfavorable Prostate Cancer	Favorable Prostate Cancer	*P*-value
No. of patients	2548	235	
Age (yr.)	65 (49–77)	63 (43–77)	0.002
Body mass index (kg/m^2^)	24.6 ± 2.9	24.4 ± 2.7	0.671
PSA (ng/ml)	9.49 ± 8.88	6.92 ± 10.99	<0.001
Prostate volume (cc)	32.1 ± 13.6	40.8 ± 22.4	<0.001
Transitional zone volume (cc)	14.6 ± 6.6	18.7 ± 10.7	<0.001
PSAD (ng/ml/cc)	0.296 ± 0.232	0.170 ± 0.087	<0.001
TzPSAD (ng/ml/cc)	0.650 ± 0.572	0.370 ± 0.162	<0.001
ETzD (ng/ml/cc)	0.252 ± 0.168	0.102 ± 0.097	<0.001
**Surgical T stage**			
<T3	2263 (88.8%)	235 (100%)	<0.001
≥T3	285 (11.2%)	—	
**Surgical N stage**			
<N1	2121 (83.2%)	235 (100%)	<0.001
≥N1	427 (16.8%)	—	
**Surgical Gleason score**			
<6	349 (13.7%)	235 (100%)	<0.001
≥7	2199 (86.3%)	—	

PSA; prostate specific antigen, PSAD; PSA Density, TzPSAD; transitional zone PSAD, ETzD; Extra-Transitional zone Density.

## Discussion

Serum PSA level remains the most common reference marker used to screen for prostate cancer, but it has limited ability to predict malignancy, as expressed in terms of sensitivity and specificity. To improve sensitivity, clinical guidelines^3^ recently recommended reducing the PSA cutoff to 2.5 ng/ml rather than the traditional threshold of 4.0 ng/mL because the latter has a sensitivity of 67–80%, which implies that some cancers are missed with the conventional cutoff^4^. However, given the trade-offs between sensitivity and specificity, adjusting the cutoff level has limited ability to enhance PSA resolution as an oncologic hazard predictor.

Previous efforts to improve PSA predictive value have included stratifying the influence of size-based structural changes by prostate enlargement using PSAD^5^, transitional zone PSA density^6^, and nomograms^7–9^. Some efforts have demonstrated improvement for prediction of prostate malignancy detection^7–9^ and identification of suitable candidates for active surveillance^10,11^. Only a few nomograms are utilized in general practice for limited purposes, primarily because they lack appropriate external validation or calibration to compensate for the weaknesses of limited size and generality^12–16^. Ironically, in contrast to modern nomograms with limited performance developed based on logistic regression modelling with assumption of linearity between the size and oncological hazard, classic PSAD, the so-called “comeback kid”^17^, reflects an inverse rather than linear relationship and has become generalized enough to be considered a general classification criterion for identifying active surveillance candidates^18^. ETzD was developed as an upgraded version of PSAD or TzPSAD based on non-linear regression models that reflect not only the inverse but also linear influence of PSA and size parameters on oncological hazard. ETzD provides more sophisticated clinical rationale to guide the tough decision of whether to perform a prostate biopsy in patients with an enlarged prostate and moderately elevated PSA, not only estimating PSA decrease before transurethral prostate surgery but also determining the personalized PSA cut-off value for patients with stromal hyperplasia.

Because ETzD predicts oncological hazard by stratifying the influence of enlarged transitional zone, the stratification benefit over other classic parameters could be more significant for prostates with larger TzS compared to PS. This inherent characteristic could be one reason for failure to demonstrate a definitive superiority of ETzD compared to PSAD or TzPSAD in the performance analysis predicting prostate cancer aggressiveness. Previously diagnosed prostate cancer patients have an elevated serum PSA level mainly due to prostate cancer, and so the contribution of transitional enlargement should be smaller than in the general population. Therefore, in this situation, the benefit of ETzD over PSAD could be relatively low, although the performance was better than PSA and comparable with PSAD.

The performance analyses were intended to serve as representative evaluations of the utility of this novel parameter as a substitute for conventional PSA with appropriate validation. From the perspective of statistical technique, DeLong test was applied for the comparison of predictive performance. There exists a concern that DeLong method should not be utilized to compare ROC-AUC curve of nested models, especially the regression models against the individual variables used to construct the predictor, because in the process of nesting variables, the distribution of the variable could be changed and normality could be destructed^19^. However, all of ETzD, PSAD and TzPSAD were not developed based on the direct regression model between the predictors and predicted outcome like other nested model. To reflect PSA kinetics according prostatic hyperplasia, the indirect and intrinsic regression was partly included into a part of the hierarchical modelling during the development process of ETzD. Courtesy of the hierarchical modelling with pathophysiological hypothesis, we observed that the distribution and normality could be preserved for ETzD during development process. Such a phenomenon, the preservation of distribution and normality has been also observed at PSAD and TzPSAD, unlike with other nested model. This might be why most of previous literatures have been considered PSAD and TzPSAD not as a parameter with distrusted normality, but as a parameter with normality, similar with the case of ETzD. Therefore, we believe that DeLong method is a proper methodology to compare ROC-AUC curve between parameters in our study.

Future work will need to focus on calibrating the formula for ETzD in larger cohort representative of diverse demographics such as age and race. Likewise, additional studies are needed to suggest appropriate ETzD cut-off values for each PSA decision criterion. Finally, the benefit of additional size parameter measurements in addition to serum PSA obtained by TRUS or other imaging methodologies for the screening of prostate cancer should be evaluated using a cost-benefit analysis in the context of the utility of the ETzD parameter.

## Conclusions

We proposed ETzD as a way to quantify the oncological hazard of the prostate more precisely by stratification of PSA deviation due to BPH-induced structural changes. During performance analysis and validation, ETzD demonstrated better performance than conventional PSA, PSAD, or TzPSAD for prostate cancer detection among prostates with stromal enlargement and moderately elevated PSA. Furthermore, ETzD was more predictive of unfavorable prostate cancer than PSA and similar to PSAD or TzPSAD. In summary, we verified the utility of ETzD as a universal parameter that exhibits superior performance and may potentially be substituted for conventional parameters such as PSA, PSAD and TzPSAD.
